# Risk factors for post-transplant relapse and survival in younger adult patients with t(8;21)(q22;q22) acute myeloid leukemia undergoing allogeneic hematopoietic stem cell transplantation: A multicenter retrospective study

**DOI:** 10.3389/fonc.2023.1138853

**Published:** 2023-02-09

**Authors:** Wei Zhou, Guofeng Chen, Dan Gong, Yi Gao, Li Yu

**Affiliations:** ^1^ Central Laboratory, Shenzhen University General Hospital, Shenzhen University Medical School, Shenzhen, Guangdong, China; ^2^ Guangdong Key Laboratory for Biomedical Measurements and Ultrasound Imaging, National-Regional Key Technology Engineering Laboratory for Medical Ultrasound, School of Biomedical Engineering, Shenzhen University Medical School, Shenzhen, Guangdong, China; ^3^ Department of Endoscopy, National Clinical Research Center for Cancer, Key Laboratory of Molecular Cancer Epidemiology of Tianjin, Key Laboratory of Cancer Prevention and Therapy, Tianjin’s Clinical Research Center for Cancer, Tianjin Medical University Cancer Institute and Hospital, Tianjin, China; ^4^ Department of Hematology, Chinese PLA No. 965 Hospital, Jilin, China; ^5^ School of Biomedical Engineering, Shenzhen University Medical School, Shenzhen, Guangdong, China; ^6^ Department of Hematology and Oncology, International Cancer Center, Shenzhen Key Laboratory of Precision Medicine for Hematological Malignancies, Shenzhen University General Hospital, Shenzhen University Clinical Medical Academy, Shenzhen University Medical School, Shenzhen, Guangdong, China

**Keywords:** *RUNX1/RUNX1T1*, allogeneic hematopoietic stem cell transplantation, acute myeloid leukemia, relapse, minimal residual disease, t(8;21)

## Abstract

**Background:**

Outcomes of patients with t(8;21)(q22;q22) acute myeloid leukemia (AML) after allogeneic hematopoietic stem cell transplantation (allo-HSCT) remain heterogeneous.

**Methods:**

To identify the risk factors for relapse and survival after allo-HSCT in t(8;21) AML patients, we retrospectively evaluated the clinical and prognostic information of 142 patients with t(8;21) AML undergoing allo-HSCT between January 2002 and September 2018 at 15 hematology research centers in China.

**Results:**

Twenty-nine patients (20%) relapsed after undergoing allo-HSCT. A > 1-log reduction in *RUNX1/RUNX1T1*-based minimal residual disease (MRD) directly before allo-HSCT and a > 3-log reduction within the first 3 months after allo-HSCT were associated with a significantly lower post-transplant 3-year cumulative incidence of relapse (CIR, 9% vs. 62% and 10% vs. 47%,all *P* < 0.001), whereas transplantation during the second complete remission (CR2, 39% vs. 17% during CR1, *P* = 0.022), during relapse (62% vs. 17% during CR1, *P* < 0.001) and *KIT D816* mutations at diagnosis (49% vs. 18%, *P* = 0.039) were related to a significantly higher 3-year CIR. Multivariate analysis demonstrated that a > 1-log reduction in MRD directly before transplantation (CIR: hazard ratio(HR), 0.21 [0.03–0.71], *P* = 0.029; overall survival (OS): HR = 0.27 [0.08–0.93], *P* = 0.038) and a > 3-log reduction in post-transplant MRD within the first 3 months (CIR: HR = 0.25 [0.07–0.89], *P* = 0.019; OS: HR = 0.38 [0.15–0.96], *P* = 0.040) were independent favorable prognostic factors, and transplantation during relapse (CIR: HR = 5.55 [1.23–11.56], *P* = 0.041; OS: HR = 4.07 [1.82–20.12], *P* = 0.045) were independent adverse prognostic factors for post-transplant relapse and survival in patients with t(8;21) AML.

**Conclusion:**

Our study suggests that for patients with t(8;21) AML undergoing allo-HSCT, it would be better to receive transplantation during CR1 with a MRD directly before transplantation achieving at least 1-log reduction. MRD monitoring in the first 3 months after allo-HSCT might be robust in predicting relapse and adverse survival after allo-HSCT.

## Introduction

Acute myeloid leukemia (AML) with chromosomal translocation t(8;21)(q22;q22) is a common type of AML that occurs in approximately 7–8% of adult patients with AML (1, 2). Although patients with t(8;21) AML have been reported to have favorable prognosis, the outcomes for these patients remain heterogeneous. Approximately 30–40% of patients relapse after achieving complete remission (CR) (3–5). Furthermore, additional gene mutations (especially *KIT* mutations), other cytogenetic abnormalities, and several clinical features such as age and white blood cell counts at diagnosis, have been identified to be associated with a high risk of relapse and poor survival in t(8;21) AML patients (4, 6). Several studies have demonstrated that allogeneic hematopoietic stem cell transplantation (allo-HSCT) can significantly improve the outcomes of high-risk patients with t(8;21) AML (7). However, post-transplant relapse still occurs in approximately 10–20% of patients who have received allo-HSCT, thus resulting in poor survival outcomes (8, 9). Therefore, it is important to determine potential risk factors for relapse and survival after allo-HSCT in patients with t (8;21) AML.

Owing to the relatively lower application rates of allo-HSCT in t(8;21) AML, there have been few studies on the prognostic factors after allogeneic transplantation in t(8;21) AML. To date, several studies have consistently reported that the minimal residual disease (MRD) status after allo-HSCT, detected *via RUNX1-RUNX1T1* transcript levels, might be a powerful biomarker for predicting post-transplant relapse (8, 10). However, some discrepancies remain in research results from different clinical centers. Whether pre-transplant MRD measurements immediately before allo-HSCT or *KIT* mutations can predict relapse after transplantation remains controversial (8, 10). Therefore, we conducted a multicenter retrospective study to identify potential risk factors for relapse and survival after allo-HSCT in patients with t(8;21) AML.

## Materials and methods

### Patients

A total of 651 patients with t(8;21) AML, who were diagnosed between January 2002 and September 2018, were retrospectively collected from 15 AML study groups in China, as described in our previous studies (11, 12). The exclusion criteria for this study were as follows: (1) 37 patients who had no treatment information; (2) 54 patients aged < 14 years or > 60 years; (3) 418 patients who did not receive allo-HSCT. Finally, 142 consecutive patients with t(8;21) AML aged 14–60 years who underwent allo-HSCT were included in this study. This study was conducted in accordance with the principles of the Declaration of Helsinki and the Institutional Review Board guidelines of the participating institutions.

### Pre-transplant treatment

All patients received 1–2 cycles of the standard ‘7 + 3’ induction chemotherapy regimen and achieved the first CR (CR1). The post-remission consolidation therapy was either single intermediate- to high-dose cytarabine or cytarabine combined with an anthracycline. The patients received at least two cycles of consolidation therapy before undergoing allo-HSCT. The details of the therapeutic protocols have been reported previously (11). Patients who met the following criteria were recommended for allo-HSCT: (1) *KIT* mutations at diagnosis, (2) bone marrow relapse, and (3) MRD reduction of < 3-log after 2–4 cycles of consolidation chemotherapy.

### Conditioning regimens and graft-versus-host disease (GVHD) prophylaxis

Conditioning regimens were classified as myeloablative (MAC) or reduced-intensity (RIC) as recommended (13). MAC primarily included modified busulfan (1 mg/kg/6h × 3 days) + cyclophosphamide (50 mg/kg/day × 2 days), and total body irradiation (TBI) of 800–1000 cGy over one or two doses + cyclophosphamide (50 mg/kg/day × 2 days). RIC primarily included fludarabine at 30 mg/m^2^/d for 5 days + busulfan at 3.2 mg/kg/d for 2 days or reduced-dose TBI.

### *KIT* mutation screening and MRD monitoring

*KIT* gene mutations in exons 17 and 8 were detected using direct sequencing. MRD was monitored *via RUNX1/RUNX1T1* and *ABL* transcripts levels, which were quantified using TaqMan-based real-time quantitative reverse transcription polymerase chain reaction (RT-PCR) according to the recommendations of the Europe Against Cancer program (14). MRD was regularly assessed after every cycle of chemotherapy, directly before transplantation and serially at the 1, 2 and 3, 6, 9, 12, 24, 36, and 60 months after allo-HSCT. The MRD log reduction were calculated with the pretreatment *RUNX1/RUNX1T1* level at diagnosis as baseline. In this study, the pre-transplant MRD was defined as the MRD assessed directly before allo-HSCT.

### Statistics

The primary endpoint of interest was cumulative incidence of relapse (CIR) after transplantation. Event-free survival (EFS) and overall survival (OS) following transplantation were secondary endpoints of interest. The CIR, EFS, and OS were calculated from the date of stem cell reinfusion. Survival probabilities were determined using Kaplan-Meier plots, and a log-rank test was used to assess the differences in EFS and OS between groups. Gray’s method was used to assess differences in CIR (15). Univariate and multivariate analyses for EFS and OS were performed using the Cox proportional hazards regression model (16) and a Fine-Gray proportional hazards model was used to estimate the CIR. The multivariate model was built by backward selection of significant factors at *P* < 0.10 in the univariate analysis. Statistical significance was defined as a two-sided *P* value of < 0.05. Statistical analyses were conducted using Stata Statistical Software, version 15.1 (StataCorp) and R (version 3.3.3).

## Results

### Clinical characteristics of the patients

**Table 1** summarizes the clinical characteristics at diagnosis and transplant-related events of 142 t(8;21) AML patients who underwent allo-HSCT. The median age of the patients at diagnosis was 30 years (range: 14–54 years). The median follow-up time after allo-HSCT was 31.2 months (range: 0.9–135.6 months). Twenty-nine patients (20%) relapsed after allo-HSCT. The median relapse time after allo-HSCT was 6.4 months (range: 0.7–88.1 months) in the 29 relapsed patients. The 3-year CIR, EFS, and OS rates were 21% (95% confidence interval (CI): 15–29%), 63% (95% CI: 54–71%), and 69% (95% CI: 60–76%), respectively.

**Table 1:** The clinical characteristics at diagnosis and transplant-related events of 142 patients undergoing allo-HSCT.

Factor	n = 142
Age, median (range)	30 (14, 54)
Sex	
Male	86/142 (60.6%)
Female	56/142 (39.4%)
WBC, × 10^9^/L	9.6 (1.0, 68.0)
HB (g/L), median (range)	75 (20, 138)
PLT (×10^9^/L), median (range)	30 (2, 170)
Blasts in BM (%)	46 (7, 91)
Karyotype	
Sole t(8;21)	41/103 (39.8%)
Additional abnormalities other than t(8;21)	62/103 (60.2%)
*KIT* mutation	
Negative	66/91 (72.5%)
Positive	25/91 (27.5%)
D816	12/25 (48.0%)
Others	5/25 (20.0%)
Unknown	8/25 (32.0%)
Time from diagnosis to transplant, months	
Median (range)	7 (2, 67)
<6	62/142 (43.7%)
6 - 12	56/142 (39.4%)
>12	24/142 (16.9%)
Disease status prior to transplantation	
CR1	116/142 (81.7%)
CR2	18/142 (12.7%)
Relapse	8/142 (5.6%)
Conditioning regimen	
MAC	98/107 (91.6%)
RIC	9/107 (8.4%)
Type of donor	
HLA-identical sibling	62/142 (43.7%)
HLA-mismatched related	61/142 (42.9%)
HLA-matched unrelated	10/142 (7.1%)
HLA-mismatched unrelated	9/142 (6.3%)
Graft type	
BM	22/142 (15.5%)
PB	90/142 (63.4%)
PB + BM	30/142 (21.1%)

WBC, white blood cell counts; HB, hemoglobin; PLT, platelets; BM, bone marrow; CR1, first complete remission; CR2, second complete remission; MAC, myeloablative conditioning; RIC, reduced intensity conditioning; HLA, human leukocyte antigen.

### Undergoing allo-HCST in CR2 and relapse are associated with worse transplantation outcomes

**Figure 1:**
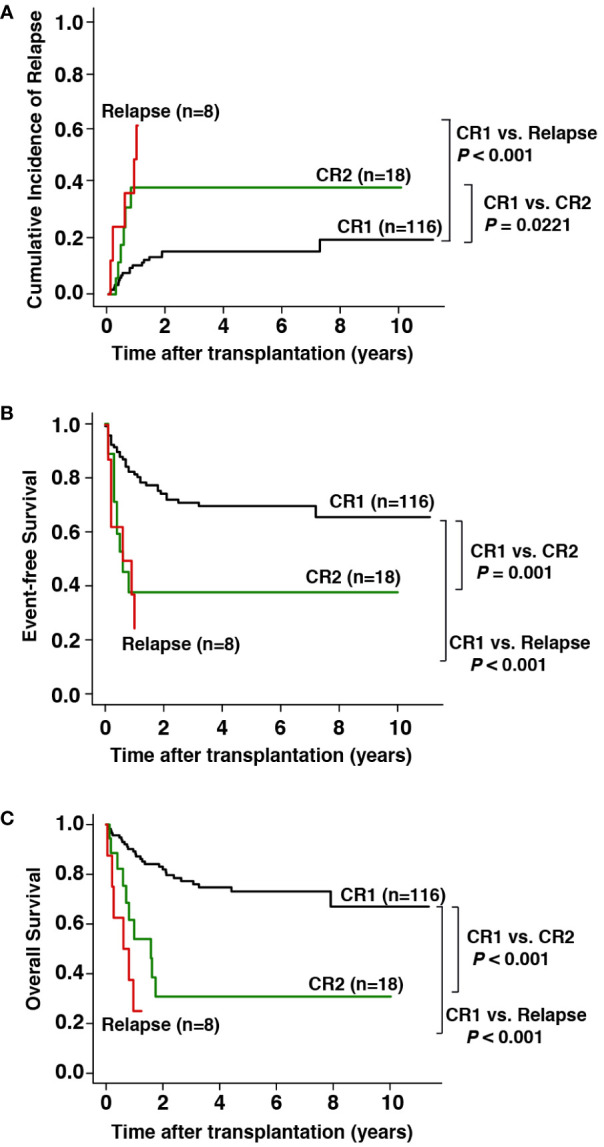
Prognostic impact of disease status at transplantation on outcomes. **(A)** Cumulative incidence of relapse (CIR), **(B)** event-free survival (EFS), and **(C)** overall survival (OS).

Overall, 116 patients (81.7%) underwent allo-HSCT during CR1, 18 (12.7%) during the second CR (CR2), and 8 (5.6%) during relapse (**Table 1**). Compared with patients who underwent allo-HSCT in CR2 and relapse, patients who underwent allo-HSCT in CR1 had a significantly lower incidence of post-transplant relapse (3-year CIR, 17% vs. 39% and 62%, respectively, *P* < 0.001; **Figure 1A**), better EFS (3-year EFS, 71% vs. 38% and 24%, respectively, *P* < 0.001; **Figure 1B**), and better OS (3-year OS, 78% vs. 31% and 25%, respectively, *P* < 0.001; **Figure 1C**).

### The role of pre-transplant MRD directly before allo-HSCT in predicting post-transplant relapse

**Figure 2:**
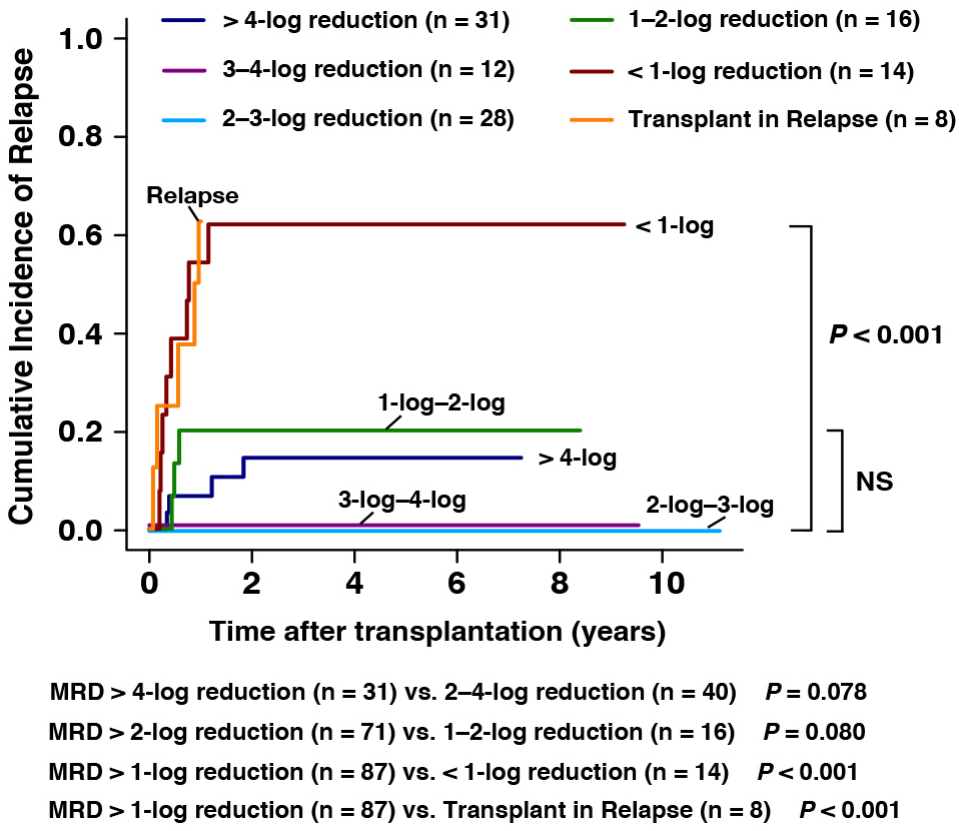
CIR rates of the patients grouped by the MRD log reduction directly before allo-HSCT.

To explore the prognosis of pre-transplant MRD for the outcomes after allo-HSCT, 109 patients with data of MRD directly before transplantation were involved in this analysis. For the patients achieving > 4-log (n = 31), 3-log to 4-log (n = 12), 2-log to 3-log, 1-log to 2-log (n =16), and <1-log (n = 14) MRD reduction assessed directly before transplantation, the 3-year CIR were 17%, 0%, 0%, 20%, and 61%, respectively (**Figure 2**). The estimated CIR showed no significant differences among patients with MRD > 1-log reduction (MRD > 4-log reduction (n = 31) vs. 2–4-log reduction (n = 40), 14% vs. 0%, *P* = 0.078; MRD > 2-log reduction (n = 71) vs. 1–2-log reduction (n = 16), 6% vs. 20%, *P* = 0.080, **Figure 2**). However, patients with pre-transplant MRD <1-log reduction (n = 14, 62% vs. 9%, *P* < 0.001) and patients in relapse (n = 8, 63% vs. 9%, *P* < 0.001) were both associated with significantly higher CIR than those with pre-transplant MRD > 1-log reduction (n = 87, **Figure 2**). We noticed that among patients with pre-transplant MRD > 4-log reduction, four patients experienced post-transplant relapse. However, it is noteworthy that all of them experienced extramedullary relapse (EMR) instead of bone marrow relapse (BMR). And two of them had extramedullary infiltration at the time of diagnosis. This might suggest that the status of pre-transplant MRD might not be strongly associated with the post-transplant EMR.

### A > 3-log reduction in MRD within the first 3 months after allo-HSCT may predict post-transplant relapse

**Figure 3:**
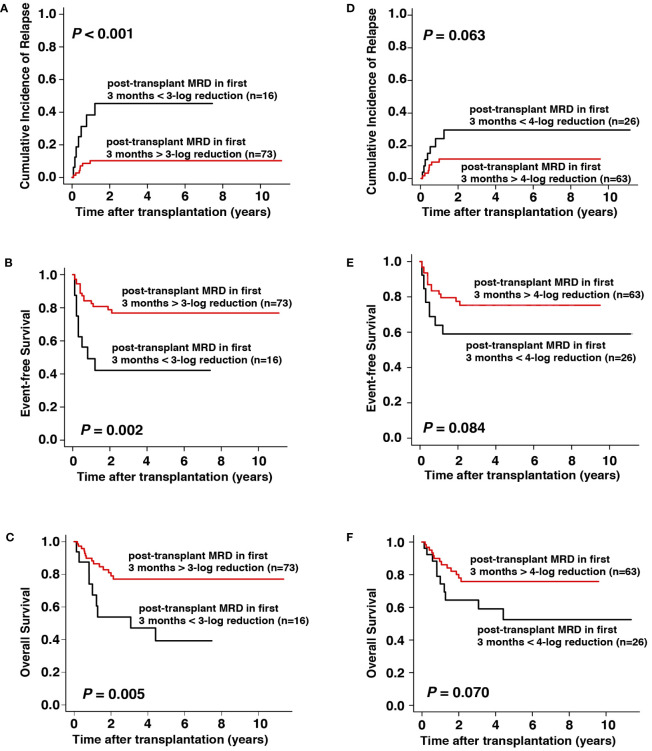
Prognostic impact of MRD within the first 3 months after allo-HSCT on outcomes. **(A–C)** CIR, EFS, and OS with cut-off value set at 3-log reduction, **(D–F)** CIR, EFS, and OS with cut-off value set at 4-log reduction.

The value of MRD monitoring after transplantation was further explored in 89 patients with complete data of MRD within the first 3 months after transplantation. We found that patients achieving MRD > 3-log reduction at each of the first three months after transplantation (n = 73) had significantly lower CIR (3-year CIR, 10% vs. 47%, respectively, *P* < 0.001), better EFS (3-year EFS, 77% vs. 42%, respectively, *P* = 0.002), and better OS (3-year OS, 78% vs. 47%, respectively, *P* = 0.005) than those who did not achieve > 3-log reduction at least once during the first 3 months after transplantation (n = 16, **Figures 3A–C**). For the 16 patients with a < 3-log MRD reduction in the first 3 months after all-HSCT, 8 of them (50.0%) received donor lymphocyte infusions (DLI) and 3 of them (18.7%) received azacytidine as interventional therapy. Seven (7/16, 43.7%) of the 16 patients relapsed during the follow-up period, among whom 1 had received DLI and 1 received azacytidine. When the cut-off value was set at 4-log reduction, the results demonstrated that patients with MRD > 4-log reduction (n = 63) showed slightly, but not significantly, lower CIR (3-year CIR, 12% vs. 30%, respectively, *P* = 0.063), better EFS (3-year EFS, 75% vs. 59%, respectively, *P* = 0.084), and better OS (3-year OS, 77% vs. 58%, respectively, *P* = 0.070) than those without > 4-log reduction (n = 26, **Figures 3D–F**).

### Patients with *KIT*-D816 mutations is likely to be correlated with worse transplantation outcomes

**Figure 4:**
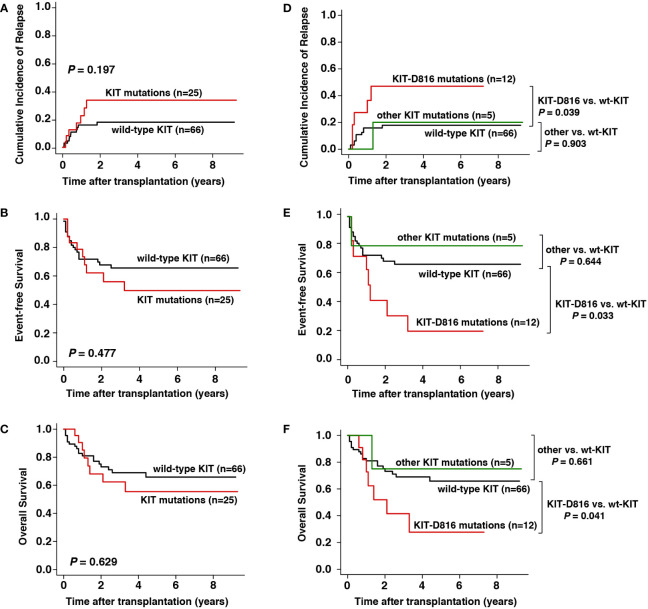
Prognostic impact of *KIT* mutation at diagnosis on outcomes. **(A–C)** CIR, EFS, and OS between patients with and without *KIT* mutation, **(D–F)** CIR, EFS, and OS between patients with *KIT*-D816 mutation, other *KIT* mutation, and without *KIT* mutation.

Among the 91 consecutive patients screened for *KIT* mutations at diagnosis, 25 (27.5%) had *KIT* mutations, among which 12 had *KIT*-D816 mutations and 5 had other *KIT* mutations (**Table 1**). The CIR (3-year CIR, 36% vs. 19%, respectively, *P* = 0.197), EFS (3-year EFS, 56% vs. 66%, respectively, *P* = 0.477) and OS (3-year OS, 62% vs. 69%, respectively, *P* = 0.629) were not significantly different between patients with and without *KIT* mutations (**Figures 4A–C**). However, patients with *KIT*-D816 mutations were associated with significantly higher CIR (3-year CIR, 49% vs. 18%, respectively, *P* = 0.039), worse EFS (3-year EFS, 32% vs. 66%, respectively, *P* = 0.033), and worse OS (3-year OS, 42% vs. 69%, respectively, *P* = 0.041) than those without *KIT* mutations; patients with other *KIT* mutations showed no significant differences compared with patients without *KIT* mutations in CIR (3-year CIR, 20% vs. 18%, respectively, *P* = 0.903), EFS (3-year EFS, 80% vs. 66%, respectively, *P* = 0.644), and OS (3-year OS, 75% vs. 69%, respectively, *P* = 0.661, **Figures 4D–F**).

### Univariate and multivariate analysis

The univariate analyses for post-transplant CIR and EFS and OS are shown in **Table 2**. Apart from the disease status at transplantation, pre-transplant MRD, post-transplant MRD and *KIT*-D816 mutations, HLA-identical sibling donor was another favorable factor influencing EFS and OS, but not CIR (HLA-identical sibling donor vs. alternate donor, hazard risk [HR], 2.07 [1.12–3.83], *P* = 0.020 for EFS and 2.11 [1.09–4.07], *P* = 0.026 for OS). Considering that only 91 patients had information of *KIT* mutation status, and 8 of them had missing data of detailed mutation sites, the *KIT*-D816 mutation status was finally not involved as a risk factor in the multivariate models. The multivariate analyses showed that the pre-transplant MRD directly before allo-HSCT >1-log reduction, and the post-transplant MRD within the first 3 months after allo-HSCT > 3-log reduction were independent favorable factors, whereas transplantation during relapse was an independent adverse prognostic factor for post-transplant CIR, EFS, and OS in t(8;21) AML patients undergoing allo-HSCT (**Table 3**).

**Table 2:** Univariate analysis of post-transplant CIR, EFS, and OS in t(8;21) AML patients undergoing allo-HSCT.

	CIR			EFS		OS	
	HR (95% CI)	*P*-value		HR (95% CI)	*P*-value	HR (95% CI)	*P*-value
Age above median, years	0.49 (0.21–1.13)	0.116		0.83 (0.45–1.50)	0.531	0.92 (0.48–1.73)	0.796
Female	0.85 (0.40–1.82)	0.680		0.63 (0.34–1.18)	0.151	0.64 (0.33–1.24)	0.186
WBC > 20 × 10^9^/L	0.51 (0.19–1.35)	0.170		0.97 (0.52–1.85)	0.940	0.99 (0.51–1.95)	0.987
HB > 100 g/L	0.69 (0.27–1.78)	0.440		1.30 (0.69–2.46)	0.419	1.53 (0.78–3.01)	0.210
PLT > 20 × 10^9^/L	1.34 (0.57–3.17)	0.500		1.19 (0.63–2.24)	0.600	1.17 (0.60–2.30)	0.640
Blasts in BM > 60%	0.46 (0.18–1.15)	0.108		1.15 (0.63–2.08)	0.651	0.98 (0.53–1.83)	0.954
*KIT*-D816 mutations	2.64 (1.01–6.87)	**0.047**		2.73 (1.21–6.14)	**0.015**	2.72 (1.14–6.47)	**0.024**
MRD directly before allo-HSCT > 1-log reduction	0.09 (0.04–0.22)	**<0.001**		0.12 (0.06–0.25)	**<0.001**	0.17 (0.08–0.36)	**<0.001**
Post-transplant MRD in first 3 months > 3-log reduction	0.19 (0.07–0.52)	**<0.001**		0.29 (0.13–0.66)	**0.003**	0.31 (0.14–0.73)	**0.007**
Disease status at transplantation							
CR2 vs. CR1	2.84 (1.12–7.19)	**0.028**		3.58 (1.73–7.37)	**0.001**	4.26 (2.03–8.94)	**<0.001**
Relapse vs. CR1	7.35 (2.93–18.44)	**<0.001**		6.46 (2.62–15.93)	**<0.001**	7.76 (2.82–21.27)	**<0.001**
HLA-identical sibling donor (vs. Alternate donor)	1.62 (0.73–3.59)	0.240		2.07 (1.12–3.83)	**0.020**	2.11 (1.09–4.07)	**0.026**
MAC (vs. RIC)	1.35 (0.46–3.96)	0.590		1.37 (0.48–3.91)	0.553	0.32 (0.04–2.38)	0.268
II-IV aGVHD	1.10 (0.46–2.62)	0.830		0.69 (0.31–1.53)	0.361	0.84 (0.38–1.90)	0.684
cGVHD	1.61 (0.74–3.48)	0.220		1.59 (0.83–3.06)	0.165	1.24 (0.60–2.60)	0.561

CIR, cumulative incidence of relapse; EFS, event-free survival, OS, overall survival; WBC, white blood cell counts; HB, hemoglobin; PLT, platelets; BM, bone marrow; MRD: minimal residual disease; CR1, first complete remission; CR2, second complete remission; HLA, human leukocyte antigen; MAC, myeloablative conditioning; RIC, reduced intensity conditioning; aGVHD, acute graft-versus-host disease; cGVHD, chronic graft-versus-host disease; HR, hazard ratio. P-value < 0.05 is marked in bold.

**Table 3:** Multivariate analysis of post-transplant CIR, EFS, and OS in t(8;21) AML patients undergoing allo-HSCT.

	CIR		EFS		OS	
	HR (95% CI)	*P*-value	HR (95% CI)	*P*-value	HR (95% CI)	*P*-value
MRD directly before allo-HSCT > 1-log reduction	0.21 (0.03–0.71)	**0.029**	0.21 (0.06–0.78)	**0.020**	0.27 (0.08–0.93)	**0.038**
Post-transplant MRD in first 3 months > 3-log reduction	0.25 (0.07–0.89)	**0.019**	0.31 (0.12–0.81)	**0.017**	0.38 (0.15–0.96)	**0.040**
Disease status at transplantation						
CR2 vs. CR1	1.52 (0.49–4.70)	0.460	1.69(0.38–7.42)	0.485	2.90 (0.74–11.38)	0.125
Relapse vs. CR1	5.55 (1.23–11.56)	**0.041**	3.02 (1.39–9.66)	**0.048**	4.07 (1.82–20.12)	**0.045**
HLA-identical sibling donor (vs. Alternate donor)	-	-	0.81 (0.29–2.28)	0.696	0.65 (0.25–1.70)	0.379

CIR, cumulative incidence of relapse; EFS, event-free survival, OS, overall survival; WBC, white blood cell counts; HB, hemoglobin; PLT, platelets; BM, bone marrow; MRD: minimal residual disease; CR1, first complete remission; CR2, second complete remission; HLA, human leukocyte antigen; HR, hazard ratio. P-value < 0.05 is marked in bold.

## Discussion

In this multicenter study, we retrospectively evaluated 142 t(8;21) AML patients undergoing allo-HSCT with a maximum follow-up time of over ten years. The 3-year CIR, EFS, and OS rates after allo-HSCT were 21%, 63% and 69%, respectively, which is consistent with the results of previous studies (10). The present study demonstrated that pre-transplant MRD directly before allo-HSCT > 1-log reduction and post-transplant MRD within the first 3 months > 3-log reduction were independent favorable prognostic factors, and transplantation during relapse was an independent adverse prognostic factor for post-transplant relapse and survival in patients with t(8;21) AML. In addition, transplantation in CR2 and with *KIT*-D816 mutations at diagnosis might also be adverse prognostic factors to predict relapse and survival after allo-HSCT, but might not be as robust as MRD assessment.

The value of MRD assessment *via* quantitative qRT-PCR in predicting relapse after chemotherapy has been widely reported (7, 17). Furthermore, Huang and colleagues identified a 3-log reduction of post-transplant MRD assessed in the first 3 months after allo-HSCT to be a robust biomarker for predicting relapse after allo-HSCT in t(8;21) AML (8, 10, 18). Our results also supported the prognostic value of post-transplant MRD in t(8;21) AML. And we further confirmed that, compared with 4-log reduction, 3-log reduction might be a better cut-off value for post-transplant MRD in predicting the outcomes after allo-HSCT. As for the pre-transplant MRD, its prognostic significance remains to be controversial. Qin et al. found that a pre-transplant MRD < 3-log reduction might be associated with a higher incidence of relapse after allo-HSCT in t(8;21) AML (10). Halaburda et al. reported similar results in patients with core-binding factor AML undergoing allo-HSCT during CR2 (19). Conversely, Wang et al. found no significant differences in post-transplant CIR and leukemia-free survival between patients with and without an MRD reduction of > 3-log before transplantation in patients with t(8;21) AML (8). In the present study, we found that patients with a pre-transplant MRD directly before allo-HSCT < 1-log reduction, but not 3-log reduction, showed a significantly worse post-transplant outcome. And the 3-year CIR rate of patients with a pre-transplant MRD < 1-log reduction was even as high as that of patients transplanted during relapse (62% vs. 63%, respectively). This suggests that for patients achieving CR, it would be better to further achieve at least a >1-log MRD reduction before undergoing allo-HSCT.

Additionally, we explored the prognostic role of *KIT* mutations after allo-HSCT. Two previous studies have found that *KIT* mutation at diagnosis is also an adverse prognostic factor for post-transplant relapse in t(8;21) AML (8, 10), but it may not be as strong as post-transplant MRD when analyzed in a multivariate model (8). Somewhat differently, our results showed that *KIT*-D816 mutations, rather than other types of *KIT* mutations, were associated with a significantly higher incidence of relapse and worse survival after allo-HSCT. In fact, many studies have indicated that, for patients with t(8;21) AML, *KIT*-D816 gene mutations at diagnosis, rather than other *KIT* mutations, are more likely to predict relapse after chemotherapy (20–23). Our results might further suggest the adverse prognostic role of *KIT*-D816 mutations in post-transplant relapse and survival.

Transplantation in CR1 has been widely proved to be a significant favorable prognostic factor for post-transplant relapse and survival in patients with AML (24). However, whether transplantation in CR2 affects the outcomes after allo-HSCT in t(8;21) AML patients remains debatable (25–27). Qin et al. demonstrated that, in t(8;21) AML patients, patients transplanted in CR2 trended toward a higher 3-year CIR (33.3% vs. 18.8%) compared with that in CR1, but the difference was not statistically significant (*P* = 0.071). However, the present study demonstrated that transplantation in CR2 was associated with a significantly higher risk of relapse and shorter OS after allo-HSCT in patients t(8;21) AML. Our results might further suggest the importance of rapid identification of patients with high-risk relapse and treating them with allo-HSCT or other aggressive therapies before they experience bone marrow relapse.

One limitation of our study was its retrospective nature. In addition, next-generation sequencing was performed in only a few patients; thus, we could not further explore the role of additional genetic co-mutations in t(8;21) AML patients after allo-HSCT.

In summary, our results suggest that for patients with t(8;21) AML who plan to receive allo-HSCT, it would be better to perform transplantation during CR1 with a pre-transplant MRD directly before allo-HSCT of at least >1-log reduction. MRD in the first 3 months after allo-HSCT might be robust prognostic factors for post-transplant relapse and survival in patients with t(8;21) AML. In addition, *KIT*-D816 mutations at diagnosis and transplantation in CR2 may also be useful in predicting the potential risk of relapse after allo-HSCT. These results should be investigated further in larger clinical trials.

## Data availability statement

The original contributions presented in the study are included in the article/Supplementary Material. Further inquiries can be directed to the corresponding author.

## Ethics statement

The studies involving human participants were reviewed and approved by the Ethics Committee of PLA General Hospital. The patients/participants provided their written informed consent to participate in this study.

## Author contributions

WZ drafted the manuscript and analyzed the data. WZ, GC, and DG collected the clinical data. YG provided valuable advice, LY designed the research and revised the manuscript. All authors contributed to the article and approved the submitted version.

## References

[1] Grimwade D Walker H Oliver F Wheatley K Harrison C Harrison G. The importance of diagnostic cytogenetics on outcome in AML: analysis of 1,612 patients entered into the MRC AML 10 trial. the medical research council adult and children's leukaemia working parties. Blood (1998) 92(7):2322–33. doi: 10.1182/blood.V92.7.2322 9746770

[2] Swerdlow SH Campo E Harris NL Jaffe ES Pileri SA Stein H. WHO classification of tumours of haematopoietic and lymphoid tissues. Lyon, France: IARC (2008).

[3] Schlenk RF Benner A Krauter J Buchner T Sauerland C Ehninger G. Individual patient data-based meta-analysis of patients aged 16 to 60 years with core binding factor acute myeloid leukemia: a survey of the German acute myeloid leukemia intergroup. J Clin Oncol (2004) 22(18):3741–50. doi: 10.1200/JCO.2004.03.012 15289486

[4] Dohner H Estey E Grimwade D Amadori S Appelbaum FR Buchner T. Diagnosis and management of AML in adults: 2017 ELN recommendations from an international expert panel. Blood (2017) 129(4):424–47. doi: 10.1182/blood-2016-08-733196 PMC5291965 27895058

[5] Marcucci G Mrozek K Ruppert AS Maharry K Kolitz JE Moore JO. Prognostic factors and outcome of core binding factor acute myeloid leukemia patients with t(8;21) differ from those of patients with inv(16): a cancer and leukemia group b study. J Clin Oncol (2005) 23(24):5705–17. doi: 10.1200/JCO.2005.15.610 16110030

[6] Papaemmanuil E Gerstung M Bullinger L Gaidzik VI Paschka P Roberts ND. Genomic classification and prognosis in acute myeloid leukemia. New Engl J Med (2016) 374(23):2209–21. doi: 10.1056/NEJMoa1516192 PMC4979995 27276561

[7] Zhu HH Zhang XH Qin YZ Liu DH Jiang H Chen H. MRD-directed risk stratification treatment may improve outcomes of t(8;21) AML in the first complete remission: results from the AML05 multicenter trial. Blood (2013) 121(20):4056–62. doi: 10.1182/blood-2012-11-468348 23535063

[8] Wang Y Wu DP Liu QF Qin YZ Wang JB Xu LP. In adults with t(8;21)AML, posttransplant RUNX1/RUNX1T1-based MRD monitoring, rather than c-KIT mutations, allows further risk stratification. Blood (2014) 124(12):1880–6. doi: 10.1182/blood-2014-03-563403 25082877

[9] Yoon JH Kim HJ Kim JW Jeon YW Shin SH Lee SE. Identification of molecular and cytogenetic risk factors for unfavorable core-binding factor-positive adult AML with post-remission treatment outcome analysis including transplantation. Bone marrow transplantation (2014) 49(12):1466–74. doi: 10.1038/bmt.2014.180 25111512

[10] Qin YZ Wang Y Xu LP Zhang XH Chen H Han W. The dynamics of RUNX1-RUNX1T1 transcript levels after allogeneic hematopoietic stem cell transplantation predict relapse in patients with t(8;21) acute myeloid leukemia. J Hematol Oncol (2017) 10(1):44. doi: 10.1186/s13045-017-0414-2 28166825 PMC5294828

[11] Zhou W Chen G Gong D Li Y Huang S Wang N. Loss of the y chromosome predicts a high relapse risk in younger adult male patients with t(8;21) acute myeloid leukemia on high-dose cytarabine consolidation therapy: a retrospective multicenter study. Leuk Lymphoma (2020) 61(4):820–30. doi: 10.1080/10428194.2019.1683734 31724463

[12] Chen G Zhou W Gong D Li Y Huang S Wang N. Loss of X chromosome predicts favorable prognosis in female patients with t(8;21) acute myeloid leukemia. Leuk Lymphoma (2020) 61(5):1168–77. doi: 10.1080/10428194.2019.1709836 31916883

[13] Bacigalupo A Ballen K Rizzo D Giralt S Lazarus H Ho V. Defining the intensity of conditioning regimens: working definitions. Biol Blood marrow Transplant (2009) 15(12):1628–33. doi: 10.1016/j.bbmt.2009.07.004 PMC2861656 19896087

[14] Gabert J Beillard E van der Velden VH Bi W Grimwade D Pallisgaard N. Standardization and quality control studies of 'real-time' quantitative reverse transcriptase polymerase chain reaction of fusion gene transcripts for residual disease detection in leukemia - a Europe against cancer program. Leukemia (2003) 17(12):2318–57. doi: 10.1038/sj.leu.2403135 14562125

[15] Gray R. A class of K-sample tests for comparing the cumulative incidence of a competing risk. Ann Stat (1988) 16 (3):1141–54. doi: 10.1214/aos/1176350951

[16] Andersen P Gill R. Cox's regression model for counting processes: A Large sample study. Ann Statistics. (1982) 10(4):1100–20. doi: 10.1214/aos/1176345976

[17] Yin JA O'Brien MA Hills RK Daly SB Wheatley K Burnett AK. Minimal residual disease monitoring by quantitative RT-PCR in core binding factor AML allows risk stratification and predicts relapse: results of the united kingdom MRC AML-15 trial. Blood (2012) 120(14):2826–35. doi: 10.1182/blood-2012-06-435669 22875911

[18] Yan CH Liu DH Liu KY Xu LP Liu YR Chen H. Risk stratification-directed donor lymphocyte infusion could reduce relapse of standard-risk acute leukemia patients after allogeneic hematopoietic stem cell transplantation. Blood (2012) 119(14):3256–62. doi: 10.1182/blood-2011-09-380386 22337715

[19] Halaburda K Labopin M Mailhol A Socie G Craddock C Aljurf M. Allogeneic stem cell transplantation in second complete remission for core binding factor acute myeloid leukemia: A study from the acute leukemia working party of the European society for blood and marrow transplantation. Haematologica (2019) 105(6):1723–30. doi: 10.3324/haematol.2019.222810 PMC7271580 31439677

[20] Yui S Kurosawa S Yamaguchi H Kanamori H Ueki T Uoshima N. D816 mutation of the KIT gene in core binding factor acute myeloid leukemia is associated with poorer prognosis than other KIT gene mutations. Ann hematol (2017) 96(10):1641–52. doi: 10.1007/s00277-017-3074-y 28762080

[21] Paschka P Marcucci G Ruppert AS Mrozek K Chen H Kittles RA. Adverse prognostic significance of KIT mutations in adult acute myeloid leukemia with inv(16) and t(8;21): a cancer and leukemia group b study. J Clin Oncol (2006) 24(24):3904–11. doi: 10.1200/JCO.2006.06.9500 16921041

[22] Cairoli R Beghini A Grillo G Nadali G Elice F Ripamonti CB. Prognostic impact of c-KIT mutations in core binding factor leukemias: an Italian retrospective study. Blood (2006) 107(9):3463–8. doi: 10.1182/blood-2005-09-3640 16384925

[23] Qin YZ Zhu HH Jiang Q Xu LP Jiang H Wang Y. Heterogeneous prognosis among KIT mutation types in adult acute myeloid leukemia patients with t(8;21). Blood Cancer J (2018) 8(8):76. doi: 10.1038/s41408-018-0116-1 30087318 PMC6081455

[24] Halaburda K Labopin M Mailhol A Socie G Craddock C Aljurf M. Allogeneic stem cell transplantation in second complete remission for core binding factor acute myeloid leukemia: a study from the acute leukemia working party of the European society for blood and marrow transplantation. Haematologica (2020) 105(6):1723–30. doi: 10.3324/haematol.2019.222810 PMC7271580 31439677

[25] Michelis FV Messner HA Atenafu EG McGillis L Lambie A Uhm J. Patient age, remission status and HCT-CI in a combined score are prognostic for patients with AML undergoing allogeneic hematopoietic cell transplantation in CR1 and CR2. Bone marrow transplantation (2015) 50(11):1405–10. doi: 10.1038/bmt.2015.165 26168067

[26] Hyakuna N Hashii Y Ishida H Umeda K Takahashi Y Nagasawa M. Retrospective analysis of children with high-risk acute myeloid leukemia who underwent allogeneic hematopoietic stem cell transplantation following complete remission with initial induction chemotherapy in the AML-05 clinical trial. Pediatr Blood cancer (2019) 66(10):e27875. doi: 10.1002/pbc.27875 31309713

[27] Malard F Labopin M Stuhler G Bittenbring J Ganser A Tischer J. Sequential intensified conditioning regimen allogeneic hematopoietic stem cell transplantation in adult patients with intermediate- or high-risk acute myeloid leukemia in complete remission: A study from the acute leukemia working party of the European group for blood and marrow transplantation. Biol Blood marrow Transplant (2017) 23(2):278–84. doi: 10.1016/j.bbmt.2016.11.002 27816650

